# Supplementary material for: Evaluating the Cysteine-Rich and Catalytic Subdomains of Human Tyrosinase and OCA1-Related Mutants Using 1 μs Molecular Dynamics Simulation
Source: Int J Mol Sci. 2023 Aug 22;24(17):13032. doi: 10.3390/ijms241713032 (PMC10487697; doi:10.3390/ijms241713032)
Supplement: Supplementary file 1 [file ijms-24-13032-s001.zip › ijms-2453200-supplementary.pdf]

Evaluating the Cysteine-Rich and Catalytic Subdomains of Human Tyrosinase and OCA1-related Mutants using 1µs Molecular Dynamics Simulation

Taariq Woods & Yuri V. Sergeev. National Eye Institute, National Institutes of Health, Bethesda, MD

Supplemental Materials

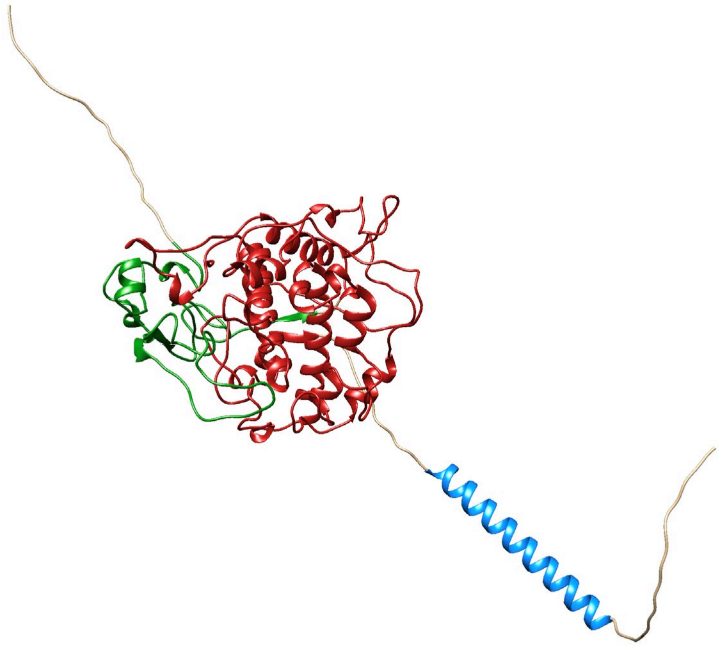

Figure S1: Domain architecture of human Tyr.
Highlighted here are the cysteine-rich subdomain (green) and catalytic subdomain (red), making up the intra-melanosomal domain, and the transmembrane helix (blue). The unstructured regions proximal to the intra-melanosomal domain and transmembrane helix are the signal peptide and cytoplasmic domain, respectively. This structure was generated from AlphaFold (https://alphafold.ebi.ac.uk).

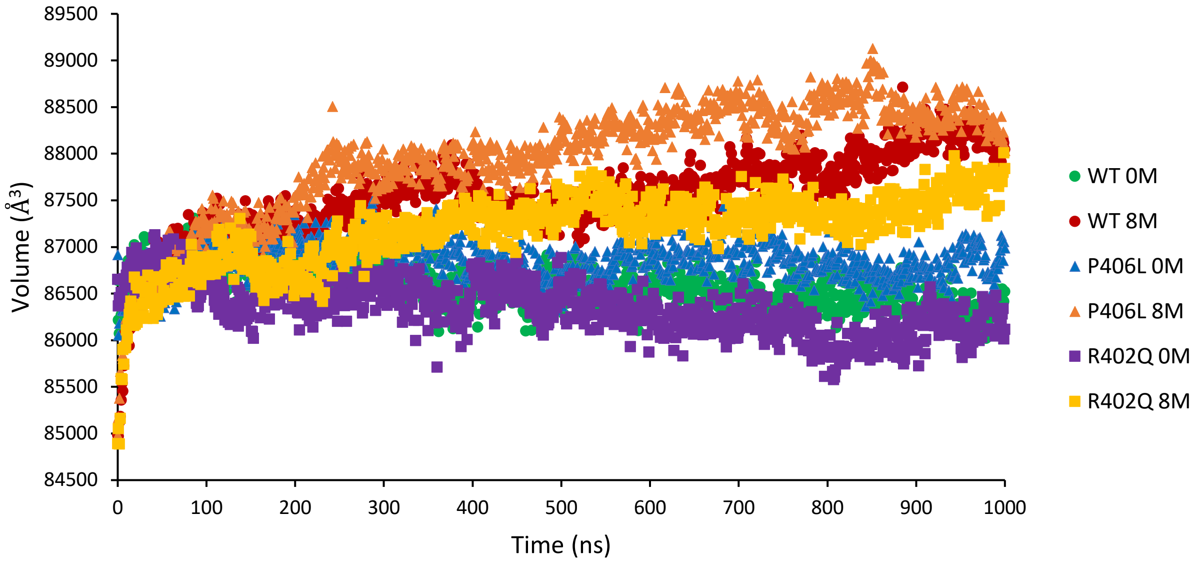

Figure S2: Comparing MD trajectories between wild type and mutants for pure water and 8M urea environments. Displayed here are the average solvent accessible volume values for the WT (circles), P406L mutant (triangles), and R402Q mutant (squares) in water and 0M urea at 1μs.

Table S1: Molecular Dynamics Resource Configuration and Efficiency.

Simulation	Total Atoms	GPU	CPU Threads	Simulated Time (μs)	Experiment Time (days)	Efficiency (ns/day)
Tyr_{tr} in Water	57724	v100x	16	1	42.83	23.35
Tyr_{tr} in 8M Urea	36084	v100x	16	1	36.36	27.5

Table S2: The urea model parameterized using the AutoSMILES program.

```
                                 AM1 CALCULATION RESULTS

**********************************************************************
*********
 *  GEO-OK    - OVERRIDE INTERATOMIC DISTANCE CHECK
 *  NOLOG     - SUPPRESS LOG FILE TRAIL, WHERE POSSIBLE
 *  NOANCI    - DO NOT USE ANALYTICAL C.I. DERIVATIVES
 *  MMOK      - APPLY MM CORRECTION TO CONH BARRIER
 *   XYZ      - CARTESIAN COORDINATE SYSTEM TO BE USED
 *  DMAX=     - TAKE MAXIMUM STEP SIZE OF 0.100 ANG/RAD
 *  CHARGE=N - NET CHARGE =  0
 *  EF        - USE EF ROUTINE FOR MINIMUM SEARCH
 *  AM1       - THE AM1 HAMILTONIAN TO BE USED
 *  PRECISE  - CRITERIA TO BE INCREASED BY 100 TIMES
 *  NOINTER  - INTERATOMIC DISTANCES NOT TO BE PRINTED
 *  EPS       - COSMO SOLVATION MODEL WITH EPSILON=78.40

**********************************************************************
*200BY200
 AM1 XYZ CHARGE=0 EF DMAX=0.1 EPS=78.4 +
 GEO-OK MMOK NOINTER NOLOG PRESS NOANCI
 Optimization in water using eigenvector following


    ATOM   CHEMICAL  BOND LENGTH    BOND ANGLE     TWIST ANGLE
   NUMBER  SYMBOL    (ANGSTROMS)     (DEGREES)       (DEGREES)
    (I)                 NA:I           NB:NA:I      NC:NB:NA:I      NA
NB   NC

      1      O
      2      N         2.25550  *                                  1
      3      N         2.25239  *      61.85351  *                 1
2
      4      C         1.22511  *      31.05124  *   -0.60567  *   1
2    3
      5      H         1.00994  *     145.25756  *   -0.26450  *   2
1    3
      6      H         1.00885  *      89.96953  * -179.71905  *   2
1    3
      7      H         1.01481  *      90.43183  *  179.82636  *   3
1    2
      8      H         1.01655  *     145.50661  *   -1.64847  *   3
1    2


          CARTESIAN COORDINATES

    NO.       ATOM         X          Y          Z
```

1	O	0.0000	0.0000	0.0000
2	N	2.2555	0.0000	0.0000
3	N	1.0625	1.9860	0.0000
4	C	1.0496	0.6319	0.0067
5	H	3.0854	0.5755	-0.0027
6	H	2.2550	-1.0088	-0.0049
7	H	0.1714	2.4715	-0.0031
8	H	1.9651	2.4533	0.0166

MOLECULAR POINT GROUP : C1

H: (AM1): M.J.S. DEWAR ET AL, J. AM. CHEM. SOC. 107 3902-3909 (1985)
C: (AM1): M.J.S. DEWAR ET AL, J. AM. CHEM. SOC. 107 3902-3909 (1985)
N: (AM1): M.J.S. DEWAR ET AL, J. AM. CHEM. SOC. 107 3902-3909 (1985)
O: (AM1): M.J.S. DEWAR ET AL, J. AM. CHEM. SOC. 107 3902-3909 (1985)

RHF CALCULATION, NO. OF DOUBLY OCCUPIED LEVELS = 12

DIAGONAL MATRIX USED AS START HESSIAN

Cycle = 1, Gradient = 125.551, Formation energy = -56.787 kcal/mol
Cycle = 2, Gradient = 76.190, Formation energy = -60.899 kcal/mol
Cycle = 3, Gradient = 44.361, Formation energy = -63.323 kcal/mol
Cycle = 4, Gradient = 63.054, Formation energy = -63.943 kcal/mol
Cycle = 5, Gradient = 40.465, Formation energy = -65.014 kcal/mol
Cycle = 6, Gradient = 34.748, Formation energy = -65.224 kcal/mol
Cycle = 7, Gradient = 19.221, Formation energy = -65.244 kcal/mol
Cycle = 8, Gradient = 15.575, Formation energy = -65.286 kcal/mol

AutoSMILES force field parameter assignment report
==
Residue UNK A 1 : Parameters for 8 atoms newly derived using AutoSMILES/GAFF/AM1BCC.
Residue UNK B 1 : Parameters for 8 atoms copied from identical residue 'UNK' parameterized with AutoSMILES/AM1BCC/GAFF earlier (net charge 0.00).
